# Supplementary material for: Characterization of aerosols generated by high-power electronic nicotine delivery systems (ENDS): Influence of atomizer, temperature and PG:VG ratios
Source: PLoS One. 2022 Dec 20;17(12):e0279309. doi: 10.1371/journal.pone.0279309 (PMC9767331; doi:10.1371/journal.pone.0279309)
Supplement: S1 Appendix — (DOCX) [file pone.0279309.s001.docx]

**S1 Appendix**

**Table 1: Student’s t-test results**: Gravimetric analysis (average aerosol mass) of all experimental combinations of atomizer, PG:VG ratio, and temperature for the various particle size groups. Within a row, the values in **Red** indicate the two experimental groups that are being tested for statistical significance, with all other parameters (not in **Red**) held constant. “N/A” indicates this experimental parameter was fixed. As an example, Line 1 shows statistical significance (p < 0.05) between the ultra-fine particle aerosol masses of atomizers A and B, at a PG:VG ratio of 65:35 and testing temperature of 200 °C.

| **Particle size group** | **Atomizer 1** | **Atomizer 2** | **PG:VG 1** | **PG:VG 2** | **Temperature 1 (°C)**°C) | **Temperature 2 (°C)** | **P-Values** |
| --- | --- | --- | --- | --- | --- | --- | --- |
| Ultra-fine particles | **A** | **B** | 65:35 | N/A | 200 | N/A | 0.049 |
|  | **B** | **C** | 65:35 | N/A | 300 | N/A | 0.004 |
|  | C | N/A | 65:35 | N/A | **200** | **300** | 0.020 |
| Fine particulate matter | **A** | **B** | 35:65 | N/A | 300 | N/A | 0.009 |
|  | **A** | **B** | 50:50 | N/A | 300 | N/A | 0.043 |
|  | **A** | **C** | 35:65 | N/A | 300 | N/A | 0.018 |
|  | **A** | **C** | 50:50 | N/A | 300 | N/A | 0.045 |
|  | A | N/A | 35:65 | N/A | **200** | **300** | 0.009 |
|  | A | N/A | **35:65** | **65:35** | 300 | N/A | 0.037 |
|  | A | N/A | **50:50** | **35:65** | 300 | N/A | 0.023 |
|  | A | N/A | 50:50 | N/A | 200 | 300 | 0.043 |
|  | B | N/A | 50:50 | N/A | 200 | 300 | 0.016 |
| Coarse particulate matter | **A** | **C** | 35:65 | N/A | 300 | N/A | 0.029 |
|  | A | N/A | 35:65 | N/A | 200 | 300 | 0.025 |
|  | A | N/A | **65:35** | **50:50** | 200 | N/A | 0.035 |
|  | B | N/A | 35:65 | N/A | **200** | **300** | 0.040 |
|  | B | N/A | 50:50 | N/A | **200** | **300** | 0.026 |
