# Supplementary material for: Characterization of aerosols generated by high-power electronic nicotine delivery systems (ENDS): Influence of atomizer, temperature and PG:VG ratios
Source: PLoS One. 2022 Dec 20;17(12):e0279309. doi: 10.1371/journal.pone.0279309 (PMC9767331; doi:10.1371/journal.pone.0279309)
Supplement: Attachment — Submitted filename: Response to Reviewers.docx [file pone.0279309.s003.docx]

Dear Reviewer,

Please find below response to each of your comments and suggested edits below:

“In general, I am very supportive of your main conclusion that conditions that lead to the ultrafine particle generation by the ENDS have to be thoroughly investigated. At the same time while reading through your manuscript I’ve found quite a few things that need to be fixed prior to further consideration.

1) Fig 2A and in the text description – more details would be beneficial per each atomizer. From the picture it seems like all three atomizers are vertical coil type where the coil is wrapped by the wick. For this type of atomizers coil itself forms an aerosol channel. There are different configurations: single coil, triple coil, five coils. Which ones were used?

***Response:*** *The coils were same as used in previous study. For clarity we have added the reference in the subsection “Atomizers and Modules”. In addition, we have added the following description about the atomizers: “All three atomizers are vertical coils (Ni 200 with a resistance of 0.15 Ω) that wrapped by the wick. Atomizer B is a dual coil, while atomizers A and C are single coil.”*

2) Fig 6 – Very poor resolution for the X-axis labels

Please provide more clear picture, so X-axis particle size labels could be readable.

***Response****: Higher resolution image attached, see Figure6 HQ.png*

3) How did you measure the Temperature? Or just used ENDS Temperature Settings that may not be accurate?

***Response:*** *The temperature measurement approach was also kept the same as established in previous study (Dibaji, et al., 2018). For brevity the following details has also been added to the “Atomizer and modules” subsection. “The ENDS control circuitry measures the voltage, resistance and current as a function of time during operation. The temperature is determined by the resistance variation with temperature. The applied ENDS in this study utilizes the DNA 200 microchip (Evolv, LLC) a popular microprocessor that can measure and control temperature. The temperature control mode allows the user to specify the maximum coil temperature (100–300 ˚C) during operation. The accuracy of the DNA 200 in measuring and controlling the temperature was evaluated against thermocouple measurements in our previous study (Dibaji et al., 2018). In that study, the power and temperature control were set at 100 W, and 300 ˚C, respectively. Thermocouples (type K) were secured to the top and middle sections of the heating coil using thermally conductive cement and the coil temperature was recorded by thermocouples as well as the DNA 200 associated software (EScribe) during simulated puffing conditions using e-liquid composition of 65% VG, 35% PG, and 3 mg/mL of nicotine. Although thermocouple measurements showed variation in temperature along the length of the coil, there was an acceptable agreement between the average temperature recorded by thermocouples and EScribe software.”*

4) Supplemental Data Table 2: Reporting CMD with very high GSD in principle is OK. But since apparently you observed bi-modal distribution, it would be more beneficial to report CMD and GSD per both modes.

***Response:*** *We included a discussion on the multi-modal distribution, and removed S1 Table 2 and discussion of CMD. Please refer to our response to comment # 24 for further details.*

5) “aerosol mass measurements showed an increase in the overall aerosol mass of fine and coarse particles with increases in temperature and decreases in propylene glycol content”.

Propylene glycol is much more volatile than glycerol therefore you should expect significant evaporation of PG from the impactor foils during the sampling. This experimental artifact should be discussed.

***Response:*** *There are two occasions when PG may preferentially evaporate over glycerol. During analysis of size distribution, and then during gravimetric analysis. For the later, which the editor points to, since the volume of the fluid recovered on the plates is large, and surface area is not high. In addition, PG’s boiling point (188.2 ^o^C) while being lower than glycerol (290 ^o^C) is still quite high. Hence, we think the relative evaporation of PG from the collected foils is significantly different from glycerol. We have expanded to discuss this issue “limitations of the study”. We also acknowledge that this issue may require future systematic studies.*

6) You need some comparative experiments pushed vs pulled air through the ENDS to justify your technology.

***Response:*** *In our experience with prior experimental set ups both for ENDS and other areas (such as facemasks and respirators) we have found the push set up to work better in absence of 100 % sealing compared to pull set up as it forces the majority of the air to pass through region of interest which in this case are the ENDS atomizer passageways. Since we did not quantitative characterize this aspect during the study, hence we are not including any data. Validation of our set up can be found in prior publications (Guha et al., 2015, American Journal of Infection Control).*

7) Regarding presence of metals in e-cig aerosol you have just one reference, but there are more references available including Talbot group and perhaps even more valid data of Pappas group.

***Response:*** *Following reference added for metals in e-cig aerosols:* Gonzalez-Jimenez, N., Gray, N., Pappas, R. S., Halstead, M., Lewis, E., Valentin-Blasini, L., ... & Blount, B. (2021). Analysis of Toxic Metals in Aerosols from Devices Associated with Electronic Cigarette, or Vaping, Product Use Associated Lung Injury. *Toxics*, *9*(10), 240.

8) L56: “The aerosol characteristics of ENDS, such as particle size distribution and the concentration of fine and ultra-fine particulate matter”

Particle size distribution as a definition includes both: particle count and particle size. You should either say “particle size distribution” or “particle size and count”.

***Response:*** *Revised as advised*

9) L75: “Fast Mobility Particle Separators (FMPS)”

Should be Fast Mobility Particle Spectrometers (not separator). Also, Differential Mobility Spectrometer (DMS500) that utilizes the same operating principle as FMPS, should be mentioned.

***Response:*** *We apologize for this oversight. We also agree that DMS500 should be added and have added it in the same sentence. Note that we also reference articles using DMS500 in Page 21 of the manuscript (with tracked changes).*

10) L77: “techniques such as Mercer impactors or condensation particle counters in isolation”

Condensation particle counter is a fast real-time technique, but it does not measure particle size.

***Response:*** *We have now deleted the phrase from the introduction and conclusion.*

11) L97: “ultra-fine particles—which may penetrate deeper into the lung”

this is not the only one health effect of ultra-fine (nano) particles

***Response:*** *We agree with the editor and slightly expanded this section and added a review paper as reference so the reader if interested can refer to it for further details. Since our introduction is already large, we kept this discussion short.*

12) L154: “Because the preliminary results suggested that the aerosols being examined from the ENDS were too concentrated for analysis using the HR-ELPI, the aerosols were diluted with room air”

What is the HR-ELPI concentration limit?

***Response:*** *The concentration limit is available in the HR-ELPI+ manual and is likely confidential information from the instrument manufacturer. The information has been provided only for the editor based on the question posed. For the completeness of the manuscript, we have added the following sentence discussing the HR-ELPI concentration limit. “In this regard, the maximum concentration limit for the HR-ELPI is size dependent and is typically 2×10^5^ #/cm3, 3.7×10^6^ #/cm3 and 7.9×10^7^ #/cm3 at approximately 10 µm, 0.1 µm, and 10 nm, respectively.”*

13) L157: “ELPI inlet as shown in Fig 2C. There was a 6:1 dilution at the HR-ELPI inlet, followed by an additional 8:1 dilution using a Dekati diluter (Dekati Ltd., Kangasala, Finland), for a combined dilution of 48:1 (Fig 2D)”

Figure 2C shows 6 L/min and 60 L/min dilutions to the 0.94 L/min flow coming from the smoking machine. The dilutions showed at Fig 2C should result in ~1/71 dilution factor. Please clarify.

Figure 2D shows some aerosol size distribution as a function of time? Please clarify.

***Response:*** *We agree with the editor. We have now replaced the previous statement with the following calculations and sentences – “*The total dilution of the e-cigarette smoke is given by (60 + 6 + 0.94)/0.94 i.e. 71.2 where two sources of 6 L/minute and 60 L/minute enter the Dekati diluter (Dekati Ltd., Kangasala, Finland) and mix with the e-cigarette flow rate of 0.94 L/minute. Subsequently 9.89 L/minute of that total flow is sampled by the HR-ELPI and the rest is discarded. *“*

*Figure 2D caption has been expanded to provide more clarity. The previous description is now replaced with the following text “*Size distributions obtained for an Atomizer plotted across time and over the entire size regime at 200 ^o^C . Only the first five scans have been shown. The scans were obtained every 30 seconds. With each subsequent scan after the first one, the peak concentration appears to reduce. The color legend represents different ranges of concentration. For simplicity the subsequent plots shown in this manuscript have been averaged (as and when needed) and hence are not presented in the same format.*”*

14) L164: “The maximum concentration was seen during the first puff 77% of the time and occurred within the first 3 puffs 94% of the time."

It indicates instability of the system performance. Needs to be discussed.

How many puffs overall per session were taken? Same as for gravimetric analysis: 10 puffs for 200 °C, and 5 puffs for 300 °C?

*Response: There are two potential sources of instability - in the e-cigarette performance i.e. aerosol generation or in the classifier i.e. the HR-ELPI. Given the temperature fluctuations in the e-cigarette and its concomitant impact on the aerosol generation its likely that the e-cigarettes were the source of the instability. In absence of a “standardized community accepted e-cigarette” or in absence of an orthogonal comparison by another rapid scanning method which we don’t currently possess the capability for, it is not possible to assess if the source of the instability was the e-cigarette. We do not think the HR-ELPI was the source of the instability as prior to each run, and during the time of data collection, the HR-ELPI set up, including all the accessories flow controllers and meters passed stability requirements in flow rate, temperature etc. We have added the following sentence “*The variation in the concentration of the aerosols with time was likely reflection of the intrinsic variation in the aerosol generation in the e-cigarettes.” *in the next sentence.*

*The editor is correct about the puffs per session. Since the gravimetric analysis were simultaneously conducted with the physical characterization, they had the same puffs at the various temperature as the editor stated. Since this is already described in the next paragraph, we have not made any changes to address this comment.*

15) Dekati Diluter operates by “Purified pressurized dilution air flows at high speed around an ejector nozzle and causes a pressure drop which draws the sample into the diluter through the nozzle”.

Forced pressure drop is very undesirable when you deal with very fragile PG-VG based ENDS aerosol that was created by nucleation. Pressure drop may cause additional aerosol evaporation. This potential artifact should be discussed.

*Response: While we agree with the editors’ comments about potential evaporation caused by the dilution, we would like to disagree with the assertion that the evaporation would be significantly impacted by the pressure drop. The reasoning is detailed below –*

- *Short residence time – The impactor looks big but its internal volume is small and residence time inside the entire stack is about 600ms.  Particles larger than 0.155µm (where most all of the mass is in ENDS) only see absolute pressures above 700mbar and would have a maximum residence time of 560ms.  Particles smaller than 0.155µm see lower pressures (down to 40mbar absolute for the smallest 0.006µm) but have a total residence time of about 40msec in that section of the impactor.  The ELPI+ raw data signal is real-time and not effected by particles that have been previously collected in the stage and would have longer residence times.*
- *Low heat transfer – For evaporation you must have heat transfer.  Heat transfer is very low for an aerosol particle as gases conduct heat much less than solids or liquids.  The ELPI+ raw data signal is generated when airborne particles hit the plate and not those after collection on a collection plate. Therefore, events after collection do not impact the size characterization.*
- *Reference evaporation times are based on a droplet on a plate and not an aerosol particle.  Evaporation rates for liquid aerosol particles are much lower than evaporation rates you will find in tables. As mentioned previously, the surface area ratio in the plates are not high as the liquid droplets start collecting to become bigger droplets, and hence the subsequent evaporation is also reduced.*
- *Liquid volatile droplets have been reported at very small sizes in the ELPI+ and are actually measured in particle bounce studies.  Some researchers use the “bounce” of aerosol particles in the lowest stage of the ELPI+ (smallest sizes and lowest pressures) to differentiate between solid and liquid particles at that size.*
- *Another way of looking at evaporation is by considering pressure drop as equivalent dilution. The mechanism by which pressure reduction drives evaporation is through reduction of vapor partial pressure around droplet that is experiencing the pressure. This can be considered as “dilution” of the gas. The following “dilution” factors through reduction of partial pressures apply for ELPI+ impactor stages:*

| ***Stage*** | ***D50% (µm)*** | ***Pressure (kPa)*** | ***Residence Times*** | ***dilution factor*** |
| --- | --- | --- | --- | --- |
| *1* | *0.006* | *4* | *0.001* | *25.3* |
| *2* | *0.0155* | *4.14* | *0.0024* | *24.5* |
| *3* | *0.0308* | *9.73* | *0.0056* | *10.4* |
| *4* | *0.0553* | *22.38* | *0.0127* | *4.5* |
| *5* | *0.096* | *38.72* | *0.0222* | *2.6* |
| *6* | *0.157* | *68.61* | *0.0391* | *1.5* |
| *7* | *0.26* | *88.89* | *0.0505* | *1.1* |
| *8* | *0.388* | *97.17* | *0.0552* | *1.0* |
| *9* | *0.612* | *99.6* | *0.0585* | *1.0* |
| *10* | *0.961* | *100.49* | *0.0622* | *1.0* |
| *11* | *1.65* | *101* | *0.0571* | *1.0* |
| *12* | *2.5* | *101.18* | *0.0578* | *1.0* |
| *13* | *3.7* | *101.25* | *0.0736* | *1.0* |
| *14* | *5.44* | *101.3* | *0.1029* | *1.0* |
| *15* | *10.02* | *101.33* | *0* | *1.0* |

*Now these equivalent “dilution” values are not much different than in instruments that use sheath air like SMPS and EEPS, and especially as in the ELPI+ the residence time with these dilution factors is lower than in the instruments with sheath air.*

*An additional work on using the ELPI to investigate volatile particles can also be found here -* [*https://trepo.tuni.fi/handle/10024/114858*](https://trepo.tuni.fi/handle/10024/114858) *that supports the points discussed in the bullets above. We have not made any change to the manuscript as we do not think the pressure drop would influence*

16) Figure 1 has been placed after Figure 6 whereas it should be the first one.

*Response: Thank you for bringing this to our attention. At the time of writing of the revision to the manuscript the ordering of figures was double checked. During resubmission, we will make sure that the ordering is correct.*

17) L278: “Lung deposition models show strong deposition in the extrathoracic, tracheobronchial, and alveolar regions for particles in the size ranges 0.0-0.10 μm, 0.10-1.0 μm, and 1.0-10 μm, respectively [28].”

Please check this statement. For instance: extrathoracic (head) region has strong deposition for either very small particles (below 20 nm) or very large particles (~10 micrometer); alveolar deposition is below 100 nm and above 1 micron

Also, exactly the same text earlier appeared on L190.

*Response: We agree with the editor’s comments and have now deleted this sentence and have added references to support the three-part categorization by size.*

*We apologize for the oversight. The same sentence was used in both results and discussion and has now been deleted from the discussion.*

18) L290: “In fact, through numerical computations [11] [17] [36], ENDS submicron aerosols have been shown to deposit at approximately twice the density in the lung’s pulmonary regions…”

None of these references (11, 17, and 36) contain numerical computations. Please check your references.

***Response:*** *We apologize for the oversight and have deleted [11][17][36] and have only retained reference to [37] in that sentence.*

19) L304: “Atomizer A generally produced one to two orders of magnitude more ultra-fine particles than did atomizers B and C, for all PG:VG ratios”.

It requires more discussion. What is so special in the design of Atomizer A.

***Response****: We have added the following sentence to provide a reasoning behind the observation – “Atomizer A was also longer than the other two atomizers which may resulted in a greater surface contact between the heating coil and the wick/e-liquid resulting in more efficient heat transfer and higher particle generation.”*

20) L309: “To further identify trends in the particle count data, we performed a relative comparison among different particle size groups, as a percentage of the total particle count, in Fig 5 based on the data presented in Fig 3. Ultra-fine particles generally constituted more than 50% of the total concentration of all particles generated at 200 °C (Fig 5).”

Figure 5 shows aerosol mass (not particle count). Perhaps you wanted to say Fig 6?

***Response****: Yes, Figure 5 was incorrect here, Figure 4 contains the total particle count relative comparison, and has been corrected in the manuscript.*

21) L321: “The mean (+/- s.d.) ratio of aerosol mass generated at 300 °C compared to 200 °C on a per puff basis (mg/puff) is 2.70 ± 2.67, averaged across all atomizers and PG:VG ratios.”

- 2.70 mg/puff ± 2.67 mg/puff. I assume that this is the aerosol mass collected on ELPI stages. It would be very useful to see TPM data collected on the filter. Normally high power MODs generate much higher aerosol mass 30-50 mg/puff, it could be up to ~100 mg/puff. From the schematics presented on Fig 2C and from the text description it is hard to understand what portion of the sample was exhausted. ~10 L/min goes into the ELPI but in order to estimate total aerosol mass generated by ENDS per puff we need to know total flow rate and portion of the flow that was not delivered to ELPI. Assuming flow values shown on Fig 2C are correct then total flow is ~ 67 L/min, therefore ~15% of the flow goes to ELPI. Then total aerosol mass should be ~18 mg/puff, but better to verify it via TPM filter method.
- You said: “aerosol mass generated at 300 °C compared to 200 °C” but I don’t see any comparison.

*Response: The exhaust split flow and its geometry is not expected to do size selective separation which would be the main reason of uneven TPM separation during exhaust splitting. Therefore a TPM filter measurement -while would verify this- was considered unnecessary at the time these experiments were performed. Currently, because of logistical reasons we are not able to perform the new experiments recommended by the editor. We acknowledge this as a limitation of our study in the Limitations section. In addition, we agree that the gravimetric analysis needs to be corrected for dilution, and have now corrected Figure 5 to reflect that change. We have also added a sentence in the discussion about the correction for dilution.*

*Regarding the second bullet, the comparison refers to the mean ratio of aerosol mass for 300℃ versus 200℃. The units mg/puff were an error, since ratios would not have units (i.e., ratio of mass is 2.70:1 for 300℃:200℃). Units have been removed in the manuscript.*

22) L348: “Indeed, Pourchez et al. [13] found that increased VG content resulted in increased overall aerosol mass generation while, conversely, Baassiri et al. [14] found an increase in PG content resulted in increased aerosol mass generation”.

Pourchez results were obtained at unrealistically high flow rate through e-cig (500 mL/s) therefore strong evaporation of PG (resulting in aerosol mass decrease) is expected.

***Response:*** *Given that our flow rate was not as high as Pourchez et al. and yet we observed similar trends it is not clear to us that their high flow rates biased their results. Nevertheless, we have made the following change to this section by adding an additional sentence:* “Indeed, Pourchez et al. [13] found that increased VG content resulted in increased overall aerosol mass generation while, conversely, Baassiri et al. [14] found an increase in PG content resulted in increased aerosol mass generation. It should be pointed out that Pourchez et al. also conducted their study at high flow rates through the e-cigarette (500 mL/s) which may have caused preferential evaporation of PG resulting in decreased aerosol mass.”

23) L160: “Note that although the puff was 3.5 seconds long, the HR-ELPI was found to detect a large concentration of aerosol for roughly 10 seconds for every puff before returning to baseline concentration”.

This long 10 s residence time is most likely caused by Diluter. Particles once entered diluter due to diluter’s design have to spend some time inside the diluter, and it may cause additional particles evaporation (hence particle size reduction).

***Response:*** *We agree with the editor and have expanded the limitations section – “In addition, as noted before, the 3.5 s puff appears to be diffused into a 10 s puff when analyzed by the HR-ELPI. It is possible that the increased residence time at the diluter may be also cause additional evaporation of the aerosols, and preferential evaporation of PG.”*

24) If GSD is larger than 2 it means that PSD is not well characterized, and it is hard to make any conclusion about either CMD or MMD. If you observed 2 modes than it is better to characterize each mode.”

***Response:*** *We included a discussion on the multi-modal distribution, and removed S1 Table 2 and discussion of CMD. Our discussion in the main manuscript is as follows “*Furthermore, consistent with prior studies such as Floyd et al., 2018, we also observed a multi-modal size distribution. For atomizer A peaks appeared around 12 nm, 40 nm, and 200-300 nm across all PG:VG ratios (Figure 6). For atomizers B and C a bimodal distribution was observed with peaks appearing around 12 nm, and then at > 500 nm. “
